# Prevalence of Polypoidal Choroidal Vasculopathy Beyond Recalcitrant Macular Neovasculopathies in a European AMD Cohort and Therapeutic Response to Brolucizumab

**DOI:** 10.3390/jcm15041492

**Published:** 2026-02-14

**Authors:** Jan Spindler, Isabel B. Pfister, Andreas Weinberger, Justus G. Garweg

**Affiliations:** 1 Berner Augenklinik, 3007 Bern, Switzerland; 2 Pallas Kliniken AG, Pallas Klinik Olten, 4600 Olten, Switzerland; 3 Department of Ophthalmology, RWTH Aachen University, 52074 Aachen, Germany; 4 Department of Ophthalmology, Inselspital, University of Bern, 3010 Bern, Switzerland; 5 Swiss Eye Institute, 6343 Rotkreuz, Switzerland

**Keywords:** recalcitrant neovascular age-related macular degeneration (nAMD), polypoidal choroidal vasculopathy (PCV), anti-VEGF, brolucizumab, intraocular inflammation, disease control

## Abstract

**Background**: Polypoidal choroidal vasculopathy (PCV) may be underdiagnosed in Europe due to the limited use of indocyanine green angiography (ICGA), in particular, in patients with occult or poorly responsive neovascular age-related macular degeneration (AMD). Based on this, we aimed to assess the prevalence of PCV beyond clinically typical recalcitrant nAMD using OCT criteria, and to report on the anatomical and functional outcomes following a switch to brolucizumab (bro). **Methods**: This retrospective case series used recently established optical coherence tomography (OCT)-based criteria to differentiate clinically typical nAMD and PCV in eyes with recalcitrant disease requiring treatment every six weeks or less. The aim was to compare the impact of switching to bro on disease activity in patients with recalcitrant nAMD and PCV over 12 months. Descriptive statistics and subgroup comparisons were performed. Data are presented as mean ± standard deviation (SD) as well as median and interquartile ranges (IQR), since data were not normally distributed. **Results**: Of the 27 eyes examined, 16 (59.3%) presented with typical recalcitrant nAMD and 11 (40.7%) with PCV. Patients with typical nAMD were older (81.4 ± 5.7 vs. 74.7 ± 7.7 years; *p* = 0.016) and exhibited less fluid (central retinal thickness in typical nAMD: 349.3 ± 95.3 µm; in PCV: 597.1 ± 348.4 µm; *p* = 0.005). This difference could be attributed to variations in pigment epithelial detachment heights (typical nAMD: 176.5 ± 102.6 µm, PCV: 384.6 ± 284.6 µm; *p* = 0.023). Twelve months after switching to bro (159 injections), the treatment interval increased from 5.7 ± 1.8 to 12.6 ± 5.1 weeks in nAMD and from 5.5 ± 1.9 to 8.0 ± 1.5 weeks in PCV patients (at switch: *p* = 0.81; after 12 months: *p* = 0.040). Visual gains after switching were maintained in two out of three patients with intraocular inflammation (IOI). **Conclusions**: PCV is remarkably underdiagnosed and overrepresented in the group of eyes with recalcitrant nAMD. Despite the inherent risk of IOI in response to bro, these results support the potential of bro as a third-line option for patients with eyes requiring anti-VEGF treatment every 6 weeks or less, provided that patients are monitored closely.

## 1. Introduction

Clinically, advanced neovascular age-related macular degeneration (nAMD) and polypoidal choroidal vasculopathy (PCV) exhibit striking similarities, including a shared genetic background. However, there are also significant differences: patients with PCV tend to be younger and respond less reliably to anti-vascular endothelial growth factor (anti-VEGF) agents [1,2,3]. A high proportion of these patients do not respond well and require frequent treatment [4,5]. Conversely, eyes with PCV may experience greater visual improvement and favourable long-term anatomical stability [6,7]. These differences may be related to their pathophysiology: typical nAMD results from subretinal neovessel formation that does not respect tissue boundaries. In response to treatment, these neovascular membranes undergo an endothelial–mesenchymal transition, resulting in a fibrovascular scar [8]. The pathophysiology of PCV is not fully understood. Polypoidal changes in the choroidal vasculature have been linked to pachychoroid changes in the larger choroidal vasculature; however, they do not evoke endothelial cell proliferation or vascular spreading. Therefore, they may regress without fibrovascular scarring upon anti-VEGF treatment. Both diseases respond well to anti-VEGF therapy once disease activity is under control [9,10].

However, up to half of eyes with typical nAMD fail to achieve complete disease stability and require frequent intravitreal anti-VEGF treatments at 10-week intervals or less to maintain functional stability [11]. Virtually no information is available about the proportion of these eyes that meet imaging criteria for PCV [12].

Intravitreal brolucizumab (bro) has a strong effect on recalcitrant nAMD [13,14], with or without pigment epithelial detachment (PED), and on incompletely responsive PCV. It achieves higher rates of polypoidal regression than other anti-VEGF agents (78–93% vs. 42–56% after 3–12 months) [10,15,16,17]. Efficacy studies, including pivotal phase III trials such as HAWK and HARRIER, have shown that 12-weekly bro injections are non-inferior to fixed-dose aflibercept with respect to the change in best-corrected visual acuity (BCVA) from baseline to week 48 in Japanese patients with PCV [18]. However, bro carries a significant risk of intraocular inflammation (IOI; 4–21%), which can be severe and require systemic and local corticosteroid treatment to prevent severe vision loss [13,15,16,19,20,21,22]. Therefore, its use is restricted to cases that are difficult to treat. In our institution, bro has been used as a rescue therapy for eyes unresponsive to other anti-VEGF agents, to improve disease activity control [19,23].

PCV may escape clinical attention, resulting in significant underdiagnosis of the disease in Caucasian patients, as indocyanine green angiography (ICGA) is not routinely performed [6,24,25]. Therefore, we hypothesised that the prevalence of PCV is increased, particularly in cases of allegedly typical nAMD that require escalation of treatment to bro. To test this hypothesis, we retrospectively reassessed the imaging data of all patients who required anti-VEGF treatment every six weeks or more frequently, and who therefore received intravitreal bro as a third-line rescue treatment for neovascular macular pathology that did not respond well to treatment. We looked for signs of PCV using non-ICGA-based imaging criteria [6,12] to assess the prevalence of undiagnosed PCV beyond cases of recalcitrant nAMD, and to report the functional and anatomical outcomes of bro as a third-line therapy in eyes with recalcitrant typical nAMD and PCV.

## 2. Materials and Methods

In this retrospective, exploratory, consecutive case series, we included patients with recalcitrant neovascular age-related macular degeneration (nAMD), with or without imaging criteria favouring polypoidal choroidal vasculopathy (PCV), who received brolucizumab as a third-line treatment to control disease activity. All patients gave general consent for their coded data to be used for quality control and clinical research purposes, after the study was approved by the Bern Cantonal Ethics Committee (registration number 2020-01847). The study complied with Swiss federal laws, the International Council for Harmonisation E6 Good Clinical Practice Guideline, and the Declaration of Helsinki (latest version).

‘Recalcitrant nAMD’ was defined as persistent intraretinal and/or subretinal fluid on spectral-domain optical coherence tomography (SD-OCT) after at least six monthly anti-VEGF injections, or a maximum treatment interval of less than ten weeks since diagnosis, following a treat-and-extend (T&E) protocol [26]. Briefly, the T&E regimen involves an initial phase of monthly anti-VEGF injections to stabilise the disease and dry the retina. This is followed by a maintenance phase, where the treatment interval is gradually extended based on disease activity criteria, namely, a change in retinal fluid (typically every two weeks). PCV was differentiated from recalcitrant nAMD using non-ICGA-based criteria, as described by Cheung et al., including colour fundus imaging and OCT features [12].

### 2.1. Inclusion Criteria

The following inclusion criteria were applied: active recalcitrant nAMD or PCV, as defined by the presence of retinal fluid requiring intravitreal therapy with bro following ranibizumab (n = 6), aflibercept (n = 20), or faricimab (n = 1) as a third-line treatment; Snellen best-corrected visual acuity at or above 0.1 at diagnosis; prior treatment with ranibizumab, aflibercept, or faricimab (switching between agents before bro was permitted); and written consent to the use of their coded data. These criteria were applied to create a more homogeneous group and reduce confounding biases.

### 2.2. Exclusion Criteria

Exclusion criteria were refusal to grant consent for the use of the patient’s coded data; subretinal bleeding greater than one optic nerve head (ONH) diameter at diagnosis; pretreatment with photodynamic therapy; and pre-existing structural damage to the macula for any reason other than nAMD without functional potential. Patients with any systemic comorbidities that could interfere with treatment outcomes were also excluded, including any local or systemic rheumatoid disease and/or vasculitis requiring topical, intravitreal, or systemic treatment. Opacifications in the optic axis that prohibit ocular imaging and fundoscopy, as well as any intraocular surgeries and/or laser treatments (except YAG laser capsulotomy) within three months before inclusion, were excluded. Eyes switched to bro did not receive a formal loading but were initially maintained at the same interval as before the switch.

Data were collected retrospectively from electronic medical records at the following prespecified time points: at diagnosis; before the first anti-VEGF treatment (baseline); after the loading phase; six months before switching to bro; before the last anti-VEGF treatment before switching; at the time of switching; after the first bro injection; after three bro injections; and after six and twelve months of bro treatment. At each time point, Snellen best-corrected visual acuity (BCVA) was recorded. For statistical use, Snellen VA was converted to ETDRS letter scores (a BCVA of 1.0 is defined as 85 ETDRS letters).

### 2.3. Non-ICGA-Based Imaging Criteria Applied Here for PCV Diagnosis

At least two of the following three major diagnostic criteria had to be given: A. sub-RPE ring-like lesion, B. en face OCT complex RPE elevation, and/or C. sharp-peaked pigment epithelial detachment (PED) [12]. In addition to these criteria, OCT angiography (OCTA) was applied to prove the flow signal under the RPE (particularly at the ring-like lesions) [27].

Intraocular pressure, optical coherence tomography (OCT) imaging data (using a Heidelberg Spectralis device and HRA-2 software, Heidelberg, Germany), including centre point retinal thickness (CRT), central subfield thickness (CST), maximal PED height in the central 3 mm around the foveola (ETDRS grid), and the presence of subretinal fluid (SRF) and/or intraretinal fluid (IRF) were recorded, as well as adverse events. CRT and CST measurements were assessed according to a standard operating procedure after manually correcting the segmentation, if necessary.

The primary outcome measures were changes in BCVA, morphological parameters (CST, CRT, and PED height), and treatment intervals (i.e., comparing injection intervals before and after switching). The secondary outcomes included the total number of injections per eye before switching and the number of injections in the first year after switching. The proportion of eyes that switched to another drug, as well as clinically relevant side effects of interest (e.g., signs of IOI, with or without retinal vasculitis (RV) or retinovascular occlusion (RO)), were recorded. Data from patients who switched to another anti-VEGF drug were excluded from the evaluation after the switch.

### 2.4. Statistical Analysis

Descriptive statistics and subgroup comparison analyses were performed. According to the Shapiro–Wilk test, most of the data were not normally distributed. Therefore, the data are presented as the mean ± standard deviation (SD), as well as the median and interquartile range (IQR: 25–75%). A *t*-test was performed for group comparisons when the data were normally distributed; otherwise, a nonparametric Mann–Whitney U test was used. The Wilcoxon signed-rank test was used to analyse longitudinal changes in functional and morphological parameters, and the Friedman test for dependent samples was used to compare multiple time points in longitudinal analyses. Effect sizes, along with 95% confidence intervals (CIs), were used for group comparisons or comparisons over time. Missing values were not substituted or imputed; all analyses were performed using the available observations. A *p*-value of ≤0.05 was considered statistically significant. All statistical analyses were performed using the package SPSS V.23 (SPSS Inc., Chicago, IL, USA) and R (version 4.4.2; R: A language and environment for statistical computing; R Foundation for Statistical Computing, Vienna, Austria, 2024).

## 3. Results

Twenty-seven eyes from 27 patients were included, with a mean age of 78.7 ± 7.2 years (12 females [44.4%] and 15 males [55.6%]). Sixteen eyes were diagnosed with typical nAMD (59.3%), and according to imaging findings, eleven were categorised as PCV (40.7%). Subgroup analysis revealed that patients with typical nAMD were older than those with PCV (81.4 ± 5.7 years vs. 74.7 ± 7.7 years, respectively; *p* = 0.016; effect size: *d_Cohen_* = 0.76, CI: −1.83–−0.21), though no differences were observed for other baseline parameters, including gender, BCVA, and CST, besides age and CRT (Table 1). Both groups showed similar improvements in BCVA, CRT, and CST after switching to bro (Table 2).

**Figure 1:**
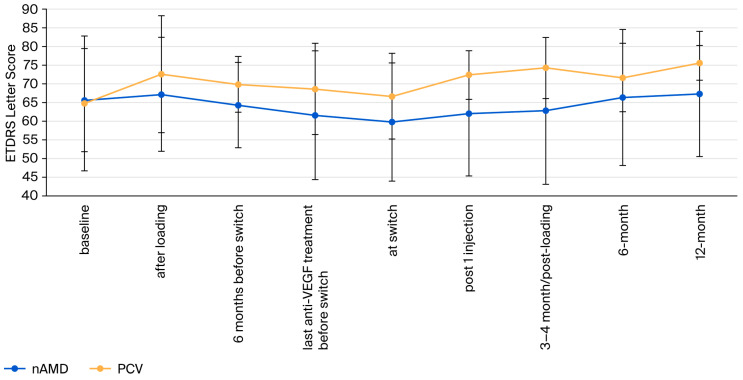
Evolution of best-corrected visual acuity (BCVA). The error bars represent standard deviations (SD).

In the pooled cohort, BCVA increased from diagnosis to the end of the loading phase, then decreased until the switch to bro. After switching, the mean BCVA recovered over 12 months (Figure 1).

The mean baseline CRT was higher in the PCV group (typical nAMD: 349.3 ± 95.3 µm; PCV: 597.1 ± 348.4 µm; *p* = 0.005; effect size: *d_Cohen_* = 0.78, CI: 0.25–1.89), while CST was more comparable in the two groups (typical nAMD: 384.5 ± 78.9 µm; PCV: 569.6 ± 326.8 µm; *p* = 0.12L; effect size: *d_Cohen_* = 0.73, CI: 0.06–1.7; see Table 2).

**Figure 2:**
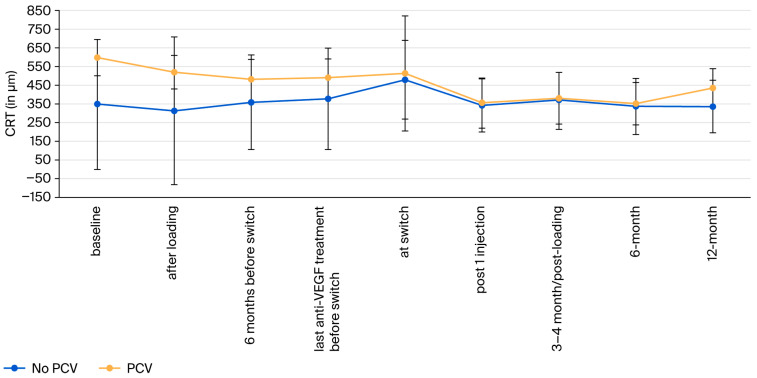
Evolution of central retinal thickness (CRT). The error bars represent standard deviations (SD).

During the loading phase, CRT improved in the pooled cohort to 383.3 ± 248.1 µm (−52.2). Thereafter, it increased again to 493.0 ± 267.2 µm (+57.5 µm). Twelve months after the switch, the CRT improved to 388.2 ± 131.1 (−104.8) µm (*p* = 0.006; effect size: dav = −0.53, CI: −2.32–−1.19; Figure 2). Similarly, CST decreased after the loading phase but increased again in both groups until the switch to bro (Table 2). After that, CST decreased again until 12 months after the switch (−75.8 µm; *p* = 0.001; effect size: dav = −0.62, CI: −2.16–1.02).

Pooled data indicate an increase in the prevalence of PED from 23 patients (85.2%) to 25 patients (92.6%) after switching to bro. Twelve months after the switch, two typical nAMD eyes (12.5%) were completely dry. Persistent PED was observed in six eyes (37.5%), while none of the PCV eyes were completely dry and ten (81.8%) showed persistent PED.

The number of injections and treatment intervals before and after the switch are displayed in Table 3 and Table 4. Pooled data showed an increase in treatment intervals after switching to bro after the third injection, accompanied by a decrease in the annual number of injections in the year after switching to bro compared to the year before switching to bro (Table 3 and Table 4). Bro treatment had to be discontinued due to IOI in three cases (11.1%). No systemic adverse events (AEs) were reported (Table 5).

## 4. Discussion

The prevalence of previously undiagnosed PCV in European cohorts of recalcitrant macular neovasculopathies has not been systematically assessed previously [28]. In our series, PCV was fivefold higher than expected in European cohorts of treatment-naïve patients with nAMD [29,30]. PCV generally responds well to anti-VEGF treatment in Caucasians [31], but complete drying of the macula is less common compared to Asian cohorts [23]. Consequently, treatment in these eyes may be challenging. Nevertheless, eyes with PCV appear to respond similarly to bro as eyes with recalcitrant typical nAMD (Figure 1 and Figure 2).

Although polyp regression is likely in both Asian and Caucasian populations [32,33], recurrence within one year is expected with as-needed (PRN) treatment [33]. None of the difficult-to-treat eyes with PCV and recalcitrant nAMD in our series qualified for PRN treatment or treatment interruption before switching. A reduced probability of complete polyp regression has been reported previously in Caucasians [34]. This needs to be independently confirmed while explaining the overrepresentation of PCV in our series. Several studies have compared anti-VEGF responses in nAMD and PCV. Our study adds to the evidence by assessing PCV prevalence in nAMD and PCV, and by comparing outcomes of bro in difficult-to-treat nAMD and PCV cases. Given the study’s limitations, we cannot provide any definitive conclusions, but the results indicate that bro may be more effective than previous anti-VEGF agents in treating recalcitrant nAMD and PCV. Published evidence and our own findings (Table 2) strongly support this, as bro seems to be highly effective in reducing retinal fluid and CRT [13,35,36,37]. However, a strong anatomic response after switching does not necessarily lead to significant visual improvement in pretreated nAMD [38]. In contrast, our PCV patients experienced a remarkable functional response, a twofold increase in treatment intervals, and a significant reduction in injections after switching to bro (Table 2, Table 3 and Table 4), resulting in relevant visual gains [19,39]. Cataract surgery was not performed to explain the visual gains in these patients.

OCT-based PCV criteria are pragmatic, though several studies have demonstrated their limited accuracy. APOIS’s three-feature OCT algorithm (sub-RPE ring-like lesion, en face RPE elevation, and sharp-peaked PED) reported 82% overall accuracy and a negative predictive value of 0.68. This means that a reasonable number of PCV cases will still be misclassified as nAMD when ICGA is not performed [12]. This has been confirmed by several other groups and two meta-analyses, which estimate a sensitivity of ~0.87 to 0.91 and a specificity of ~0.83 to 0.88 versus ICGA [40,41]. In a Caucasian nAMD cohort such as ours, an increase in prevalence from 8% with ICGA to 22% with OCT-only criteria would be expected, suggesting that ICGA may underrecognize PCV, whereas OCT may overdiagnose overlapping cases [24,42]. Three patients (11%) experienced intraocular inflammation after switching to bro, needing systemic corticosteroids in two cases. This may be justified in the face of inadequate pre-switch disease control and the absence of other treatment options. However, this must be critically discussed before switching to the treatment-experienced patients, if they accept this additional risk and can report any post-injection irregularity immediately [43].

Beyond the retrospective nature of this analysis, a clear limitation of our study is its small sample size. This can be attributed to the generally favourable response to the new generation of anti-VEGF agents, as well as the strict selection criteria for patients requiring treatment of six weeks or less during the 12 months before switching to bro, with a minimum follow-up period of 12 months. Nevertheless, we believe that the cases in this series accurately represent European cohorts of patients with recalcitrant nAMD who require intensive treatment. The effect size and confidence intervals in Supplementary Table S1 support the robustness of our findings. Furthermore, our findings are supported by those of a majority of recent reports, including randomised controlled trials and meta-analyses [13,16,19,24,29,35,37,39,40,44,45]. While our conclusions will not be precise on a quantitative level, they are unlikely to be incorrect on a qualitative level. Finally, non-ICGA-based clinical and OCT criteria have been used in a real-world setting where ICGA is not always feasible. As described by Cheung et al., these criteria demonstrate the potential for widespread use, albeit with less diagnostic accuracy (82%) than ICGA, the gold standard. Therefore, ICGA may be replaced by clinically and OCT-driven criteria in clinical routine.

## 5. Conclusions

Reclassifying OCT images using established criteria reveals that PCV is significantly overrepresented in eyes with persistent macular neovascular pathologies. Following the failure of other anti-VEGF drugs to control the disease, treatment with bro extended the treatment interval more significantly in cases of recalcitrant nAMD compared to PCV. These results support the use of bro as a third-line treatment for eyes requiring anti-VEGF treatment every six weeks or more frequently. However, early recognition and prompt treatment are essential to mitigate the risk of intraocular inflammation.

## Figures and Tables

**Table 1:** Baseline demographic findings.

Baseline Characteristics	Typical nAMD	PCV	Pooled	*p*-Value, Effect Size, 95% Confidence Interval (Typical nAMD vs. PCV)
Participants (=number of eyes), n (%)	16 (59.3)	11 (40.7)	27 (100)	
Females, n (%)	5 (31.3)	7 (63.6)	12 (44.4)	0.13 OR: 0.80 CI: 0.16–4.02
Age at baseline, (years (SD))	81.4 (5.7)	74.7 (7.7)	78.7 (7.2)	0.016 *d_Cohen_* = 0.76 CI: −1.83–−0.21
Best-corrected visual acuity (ETDRS letters (SD))	65.7 (13.8)	64.8 (18.0)	65.3 (15.2)	1.0 *d_Cohen_* = 0.52 CI: −0.83–0.71
Central retinal thickness (μm (SD))	349.3 (95.3)	597.1 (348.4)	435.5 (242.8)	0.005 *d_Cohen_* = 0.78 CI: 0.25–1.89
Central subfield thickness, (μm (SD))	384.5 (78.9)	569.6 (326.8)	448.9 (214.7)	0.12 *d_Cohen_* = 0.73 CI: 0.06–1.66

**Table 2:** Evolution of functional and anatomical outcomes.

	Typical nAMD	PCV	Pooled	*p*-Value, Effect Size, 95% Confidence Interval (Typical nAMD vs. PCV)
Best-corrected visual acuity (ETDRS letters (SD))				
Baseline (diagnosis)	65.7 (13.8)	64.8 (18.0)	65.3 (15.2)	1.0, *d_Cohen_* = 0.52, CI: −0.83–0.71
End of loading	67.2 (15.3)	72.6 (15.6)	69.1 (15.2)	0.31, *d_Cohen_* = 0.60, CI: −0.58–1.28
Switch to bro	59.9 (15.8)	66.8 (11.5)	62.7 (14.4)	0.23, *d_Cohen_* = 0.63, CI: −0.29–1.26
12 months after switch	67.4 (16.8)	75.7 (4.7)	71.5 (12.7)	0.57, *d_Cohen_* = 0.68, CI: −0.33–1.68
*p*-value (longitudinal)				
Baseline—end of loading	0.14 *d_RM_* = 0.34 CI: 0.21–1.76	0.046 *d_RM_* = 1.37 CI: 3.01–5.40	0.012 *d_RM_* = 0.63 CI: 0.93–2.20	0.08, *d_Cohen_* = 0.73, CI: −0.09–1.83
Baseline—switch to bro	0.10 *d_RM_* = −0.67 CI: −1.72–−0.20	0.80 *d_RM_* = 0.30 CI: −0.48–1.63	0.17 *d_RM_* = −0.26 CI: −0.99–0.16	0.14, *d_Cohen_* = 0.70, CI: −0.20–1.51
Switch—12 months	0.89 *d_RM_* = 0.90 CI: 0.86–2.41	0.07 *d_RM_* = 0.91 CI: 0.11–2.17	0.23 *d_RM_* = 0.88 CI: 0.61–2.06	0.15, *d_Cohen_* = 0.71, CI: −0.25–1.78
Central retinal thickness, μm (SD)				
Baseline (diagnosis)	349.3 (95.3)	597.1 (348.4)	435.5 (242.8)	0.005, *d_Cohen_* = 0.79, CI: 0.23–2.07
End of loading	314.8 (89.1)	520.3 (393.9)	383.3 (248.1)	0.13, *d_Cohen_* = 0.73, CI: −0.07–1.83
Switch to bro	479.5 (306.6)	512.6 (209.7)	493.0 (267.2)	0.27, *d_Cohen_* = 0.53, CI: −0.65–0.89
12 Months after switch	336.9 (105.6)	433.9 (140.1)	388.2 (131.1)	0.11, *d_Cohen_* = 0.71, −0.21–1.76
*p*-value (longitudinal)				
Baseline—end of loading	0.30 *d_RM_* = −0.33 CI: −1.04–0.45	0.60 *d_RM_* = −0.78 CI: −3.48–−1.30	0.052 *d_RM_* = −0.51 CI: −1.8–−0.57	0.27, *d_Cohen_* = 0.64, CI: −1.44–0.4
Baseline—switch to bro	0.033 *d_RM_* = 0.93 CI: −0.49–1.02	0.16 *d_RM_* = −0.54 CI: −2.02–−0.03	0.42 *d_RM_* = 0.2 CI: −0.42–0.74	0.12, *d_Cohen_* = 0.57, CI: −0.63–1.09
Switch—12 months	0.025 *d_RM_* = −1.16 CI: −2.72–−0.61	0.14 *d_RM_* = −1.0 CI: −3.20–−1.24	0.006 *d_RM_* = −1.05 CI: −2.55–−1.12	0.60, *d_Cohen_* = 0.57, CI: −0.70–1.22
Central subfield thickness, μm (SD)				
Baseline (diagnosis)	384.5 (78.9)	569.6 (326.8)	448.9 (214.7)	0.12, *d_Cohen_* = 0.75, CI: 0.03–1.83
End of loading	343.7 (71.3)	506.4 (367.6)	398.0 (223.7)	0.44, *d_Cohen_* = 0.70, CI: −0.18–1.69
Switch to bro	443.1 (105.5)	539.0 (181.2)	483.7 (147.3)	0.15, *d_Cohen_* = 0.68, CI: −0.12–1.48
12 Months after switch	369.4 (90.3)	442.2 (94.2)	407.9 (97.0)	0.17, *d_Cohen_* = 0.71, CI: −0.20–1.78
*p*-value (longitudinal)				
Baseline—end of loading	0.10 *d_RM_* = −0.46 CI: −1.17–0.33	0.034 *d_RM_* = −0.80 CI: −3.56–−1.68	0.013 *d_RM_* = −0.37 CI: −1.16–0.058	0.29, *d_Cohen_* = 0.63, CI: −1.38–0.45
Baseline—switch to bro	0.035 *d_RM_* = 0.65 CI: −0.26–1.21	0.58 *d_RM_* = −0.18 CI: −1.28–0.68	0.37 *d_RM_* = 0.31 CI: −0.01–1.15	0.13, *d_Cohen_* = 0.69, CI: −1.58–0.21
Switch—12 months	0.069 *d_RM_* = −1.32 CI: −3.67–−1.50	0.008 *d_RM_* = −0.83 CI: −2.20–−0.27	0.001 *d_RM_* = −0.80 CI: −1.87–−0.67	0.54, *d_Cohen_* = 0.65, CI: −1.53–0.41

*d_Cohen_* = Cohen’s d, effect size; *d_RM_* = Cohen’s d for repeated measures.

**Table 3:** Treatment intervals.

Treatment Intervals (Weeks (SD))	N	Typical nAMD		N	PCV		N	Pooled		*p*-Value (Typical nAMD vs. PCV)
Last prior to switch	15	5.7	(1.8)	10	5.5	(1.9)	25	5.6	(1.8)	0.81, *d_Cohen_* = 0.53, CI: −0.91–0.69
After third bro injection	13	8.8	(3.5)	8	7.8	(1.9)	21	8.4	(3.0)	0.75, *d_Cohen_* = 0.59, CI: −1.22–0.55
After fifth bro injection	8	10.4	(3.2)	8	7.3	(1.2)	16	8.8	(2.8)	0.038, *d_Cohen_* = 0.82, CI: −2.36–−0.21
12 months after switch to brolucizumab	8	12.6	(5.1)	7	8.0	(1.5)	15	10.5	(4.5)	0.040, *d_Cohen_* = 0.80 CI: −2.29–−0.09
*p*-value (longitudinal)										
Last prior to switch–after third bro injection		0.004 *d_RM_* = 1.49 CI: −0.07–1.66			0.035 *d_RM_* = 1.10 CI: −0.07–2.03			<0.001 *d_RM_* = 1.33 CI: 0.19–1.44		0.75 *d_Cohen_* = 0.59 *CI: 1.22–0.56*
Last prior to switch–after fifth bro injection		0.012 *d_RM_* = 3.77 CI: 1.49–4.75			0.11 *d_RM_* = 0.71 CI: −0.38–1.64			0.003 *d_RM_* = 1.83 CI: 0.55–2.20		0.07 *d_Cohen_* = 0.79 *CI: −2.17–−0.06*
Last prior to switch–12 months after switch to bro		0.012 *d_RM_* = 5.53 CI: 0.39–4.70			0.018 *d_RM_* = 1.10 CI: −0.10–2.15			0.001 *d_RM_* = 3.35 CI: 0.62–2.77		0.054 *d_Cohen_* = 0.78 *CI: −2.17–0.01*

*d_Cohen_* = Cohen’s d, effect size; *d_RM_* = Cohen’s d for repeated measures.

**Table 4:** Treatment demand (number of injections).

Number of Injections Mean (SD) Median; IQR	N	Typical nAMD	N	PCV	N	Pooled	Intergroup *p*-Value (Typical nAMD vs. PCV)
Prior to switch (baseline to switch to bro)	16	32.6 (25.6) 28; 12–46	11	39.5 (32.4) 30; 14–78	27	35.4 (27.7) 30; 12–47	0.65, *d_Cohen_* = 0.57, CI: −0.53–1.01
12 months prior to switch to bro	16	8.9 (1.9) 9.0; (7–10)	11	10.5 (2.0) 11.0; (9–12)	27	9.6 (2.0) 10.0; 8–11	0.039, *d_Cohen_* = 0.72, CI: 0.03–1.62
12 months after switch to bro	8	7.3 (2.1) 7; 6–9	7	8.0 (1.2) 8; 7–9	15	7.6 (1.7) 7; 7–9	0.41, *d_Cohen_* = 0.61, CI: −0.62–1.43
*p*-value (longitudinal)							
12 months prior to switch–12 months after switch		0.49 *d_RM_* = −0.34 CI: −1.21–0.76		0.027 *d_RM_* = −0.70 CI: −1.56–0.60		0.032 *d_RM_* = −0.73 CI: −1.32–0.16	-

*d_Cohen_* = Cohen’s d, effect size; *d_RM_* = Cohen’s d for repeated measures.

**Table 5:** Treatment discontinuation due to intraocular inflammation (IOI).

Patients who Discontinued Treatment Due to IOI	Pathology/ Complication	Number of Bro Injections Before IOI	BCVA Before IOI	BCVA After Completion of Anti-Inflammatory Therapy	Intraocular Pressure >21 mmHg	Treatment
1	Anterior uveitis with papillitis and vasculitis	5	0.25	0.25	no	Solumedrol intravenously followed by prednisone orally. Topical therapy, prednisolone acetate.
2	Severe uveitis/occlusive retinal vasculitis	2	0.5	0.6	no	Solumedrol intravenously followed by prednisone orally. Topical therapy: none.
3	Arteriovenous occlusion	5	0.32	0.16	no	Acetylsalicylate.

## Data Availability

Raw data may be requested by contact to the corresponding author upon reasonable request.

## Supplementary Materials

The following supporting information can be downloaded at: https://www.mdpi.com/article/10.3390/jcm15041492/s1, Table S1: Evolution of functional and anatomical outcomes, complementary to Table 1.
