# Supplementary material for: Prevalence of Polypoidal Choroidal Vasculopathy Beyond Recalcitrant Macular Neovasculopathies in a European AMD Cohort and Therapeutic Response to Brolucizumab
Source: J Clin Med. 2026 Feb 14;15(4):1492. doi: 10.3390/jcm15041492 (PMC12941577; doi:10.3390/jcm15041492)
Supplement: Supplementary file 1 [file jcm-15-01492-s001.zip › jcm-4103290-supplementary.pdf]

Supplemental Table S1. Evolution of functional and anatomical outcomes, complementary to Table 1.

	typical nAMD	PCV	pooled
**Best-corrected visual acuity median; IQR**			
Baseline (diagnosis)	69.9; 56.5 – 75.0	69.9; 62.0 – 75.0	69.9; 60.3 – 75.0
End of loading	73.9; 57.6 – 75.0	80.2; 69.9 – 80.2	74.4; 69.9 – 80.2
Switch to bro	60.3; 51.3 – 69.9	69.9; 60.3 – 75.0	65.1; 58.9 – 73.9
12 months after the switch	72.5; 55.9 – 80.2	73.9; 73.9 – 78.9	73.9; 69.9 – 80.2
**Central retinal thickness median; IQR**			
Baseline (diagnosis)	315; 279 – 381	487; 354 – 691	365; 305 – 480
End of loading	295; 230 – 414	450; 281 – 527	336; 244 – 438
Switch to bro	366; 329 – 523	465; 366 – 580	414; 331 – 555
12 Months after switch	297; 256 – 443	435; 306 – 556	345; 275 – 523
**Central subfield thickness, median; IQR**			
Baseline (diagnosis)	374; 312 – 405	423; 367 – 679	395; 348 – 445
End of loading	341; 275 – 424	396; 266 – 524	342; 275 – 442
Switch to bro	390; 364 – 495	530; 372 – 564	457; 368 – 563
12 Months after switch	370; 284 – 427)	450; 345 – 530	401; 327 – 505
